# Supplementary material for: Severe community-acquired pneumonia in Reunion Island: Epidemiological, clinical, and microbiological characteristics, 2016–2018
Source: PLoS One. 2022 Apr 15;17(4):e0267184. doi: 10.1371/journal.pone.0267184 (PMC9012352; doi:10.1371/journal.pone.0267184)
Supplement: Attachment — Submitted filename: PONE-D-21-05049 review.docx [file pone.0267184.s002.docx]

Severe community-acquired pneumonia in Reunion Island: Epidemiological, clinical and microbiological characteristics, 2016-2018 (Manuscript No. PONE-D-21-05049)

In their manuscript, *de Mangou et al.* present valuable data derived from a retrospective cohort of patients admitted and diagnosed with severe CAP at Reunion Island between 2016 and 2018. However, significant scarcities of methodology detailed below could be identified in the manuscript. Also, the main study results might be of more interest to the national readers of Reunion Island. In all, the Methods section needs rewriting by addition of more explicit details from the study protocol before the review process could be continued any further.

Remarks on methodology:

1. A major issue is that CAP was not defined properly (only criteria of severe CAP were stated), and details were not given on how cases of CAP were ascertained. A brief summary of in-hospital algorithms should be added to the manuscript. Also, did all the patients receive the same diagnostic (eg. imaging) and microbiological studies before the final diagnosis of CAP was arrived at?
2. What is a cytobacteriological examination? This needs further explaining.
3. Did the patients have blood cultures taken?
4. Are all patients with severe CAP hospitalized at the ICU ward at Reunion Island?
5. How were eligible patients screened and included in the final cohort?
6. Was any patient follow-up seeked during the study period? If yes, how?
7. How was data collection executed? What was the data source? How were missing data handled?
8. The primary outcome should be defined by a hard clinical end-point, eg. in-hospital all-cause mortality at day-14 or alike. "Clinical and microbiological characteristics" are not endpoints – they are characteristics.
9. There is a contradiction in the description of statistical methods: an univariate pre-screening was done (line 133), but after that, only the "most clinically relevant factors" were entered into multivariate analysis (line 135)? This needs to be elaborated.
10. Adherence to the STROBE Statement is highly encouraged during rewriting.
11. I suggest using the term bronchoalveolar lavage instead of bronchoalveolar sputum.
12. I suggest that the term "adult patients" should be emphasized.
13. The name of the American Thoracic Society should be corrected.
