# Supplementary material for: Severe community-acquired pneumonia in Reunion Island: Epidemiological, clinical, and microbiological characteristics, 2016–2018
Source: PLoS One. 2022 Apr 15;17(4):e0267184. doi: 10.1371/journal.pone.0267184 (PMC9012352; doi:10.1371/journal.pone.0267184)
Supplement: Attachment — Submitted filename: Response to Reviewers.docx.doc [file pone.0267184.s003.doc]

**COMMENTS TO AUTHOR:**

Personally, I found the following points need to be clarified:

***1. Prevalent pathogens for CAP are influenza,* S. penumoniae*. How does this compare to nosocomial pneumonia?***

We have better redefined our diagnosis criteria for CAP in the material and methods section (lines 108-112).

***2. CAP caused by influenza and* S. penumoniae *is year round, however, the authors indicated the rainfall play a role in the case of CAP.  From the figure, it appears more case for influenza CAP in dry season. Any studies on influenza CAP related to the same phenomenon?***

Several studies find an association between seasons and the occurrence of CAP and more particularly concerning influenza ([Radina P, *et al*.](https://pubmed.ncbi.nlm.nih.gov/?sort=date&term=Soebiyanto+RP&cauthor_id=26618318) Associations between seasonal influenza and meteorological parameters in Costa Rica, Honduras and Nicaragua. Geospat Health 2015 Nov 4;10(2):372/[Oyelola A et al.](https://pubmed.ncbi.nlm.nih.gov/?sort=date&term=Adegboye+OA&cauthor_id=31591495) Epidemiological analysis of association between lagged meteorological variables and pneumonia in wet-dry tropical North Australia, 2006-2016. J Expo Sci Environ Epidemiol 2020 May;30(3):448-458/ [Julian W Tang](https://pubmed.ncbi.nlm.nih.gov/?sort=date&term=Tang+JW&cauthor_id=20872724), et al. Comparison of the incidence of influenza in relation to climate factors during 2000-2007 in five countries. J Med Virol 2010 Nov;82(11):1958-65/ [Gwladys C, et al.](https://pubmed.ncbi.nlm.nih.gov/?sort=date&term=Monamele+GC&cauthor_id=29088290) Associations between meteorological parameters and influenza activity in a subtropical country: Case of five sentinel sites in Yaoundé-Cameroon. PLoS One 2017 Oct 31;12(10):e0186914/ [Kramer](https://pubmed.ncbi.nlm.nih.gov/?sort=date&term=Kramer+SC&cauthor_id=30811396) SC. Development and validation of influenza forecasting for 64 temperate and tropical countries. . PLoS Comput Biol 2019 Feb 27;15(2):e1006742/[Gentile](https://pubmed.ncbi.nlm.nih.gov/?sort=date&term=Gentile+A&cauthor_id=29596527) A, et al. Influenza virus: 16 years' experience of clinical epidemiologic patterns and associated infection factors in hospitalized children in Argentina. PLoS One 2018 Mar 29;13(3):e0195135/[Cilloniz](https://pubmed.ncbi.nlm.nih.gov/?sort=date&term=Cilloniz+C&cauthor_id=28093834) C et al. Seasonality of pathogens causing community-acquired pneumonia.  Respirology 2017 May;22(4):778-785). It has been added in the revised manuscript (lines 247-249)

***3. The mortality rate is higher for influenza CAP, is this same from other region?***

Mortality rate was higher for influenza CAP than for non-influenza CAP. The impact on mortality of the pathogen causing CAP is variable according to the studies. In Spain, Abelleira *et al*. found that CAP related to influenza A H1N1 had a poorer prognosis than non-influenza CAP ([Abelleira](https://pubmed.ncbi.nlm.nih.gov/?sort=date&term=Abelleira+R&cauthor_id=31007805) R, [Ruano-Ravina](https://pubmed.ncbi.nlm.nih.gov/?sort=date&term=Ruano-Ravina+A&cauthor_id=31007805) A,  [Lama](https://pubmed.ncbi.nlm.nih.gov/?sort=date&term=Lama+A&cauthor_id=31007805) A, [Barbeito](https://pubmed.ncbi.nlm.nih.gov/?sort=date&term=Barbeito+G&cauthor_id=31007805) G, [Toubes](https://pubmed.ncbi.nlm.nih.gov/?sort=date&term=Toubes+ME&cauthor_id=31007805) ME, [Domínguez-Antelo](https://pubmed.ncbi.nlm.nih.gov/?sort=date&term=Domínguez-Antelo+C&cauthor_id=31007805) C, [González-Barcala](https://pubmed.ncbi.nlm.nih.gov/?sort=date&term=González-Barcala+FJ&cauthor_id=31007805) FG, [Rodríguez-Núñez](https://pubmed.ncbi.nlm.nih.gov/?sort=date&term=Rodríguez-Núñez+N&cauthor_id=31007805) N, [Marcos](https://pubmed.ncbi.nlm.nih.gov/?sort=date&term=Marcos+PJ&cauthor_id=31007805) PJ, [Del Molino](https://pubmed.ncbi.nlm.nih.gov/?sort=date&term=Pérez+Del+Molino+ML&cauthor_id=31007805) ML, [Valdés](https://pubmed.ncbi.nlm.nih.gov/?sort=date&term=Valdés+L&cauthor_id=31007805) L.  Influenza A H1N1 Community-Acquired Pneumonia: Characteristics and Risk Factors-A Case-Control Study. Can Respir J 2019 Mar 17;2019:4301039. doi: 10.1155/2019/4301039. eCollection 2019) (Quah J, Jiang B, Tan PC, Siau C, Tan TY. [Impact of microbial Aetiology on mortality in severe community-acquired pneumonia.](https://pubmed.ncbi.nlm.nih.gov/30180811/) BMC Infect Dis. 2018 Sep 4;18(1):451. doi: 10.1186/s12879-018-3366-4). It has been also found that mixed viral-bacterial infections may be associated with an increased risk of mortality (Quah J, Jiang B, Tan PC, Siau C, Tan TY. [Impact of microbial Aetiology on mortality in severe community-acquired pneumonia.](https://pubmed.ncbi.nlm.nih.gov/30180811/) BMC Infect Dis. 2018 Sep 4;18(1):451. doi: 10.1186/s12879-018-3366-4)

It has been added in the revised manuscript (line 274-278).

Specific comments:

1. ***Line 54, 97 “All consecutive patients…” I am not sure consecutive is the right adj. to use.  Suggest changing to “All patients…”***

We agree with reviewer comment, it has been corrected in the revised manuscript.

2. ***Line 79 – 81, is there any CAP data in nearby area, Madagascar for example?***

There are only few studies on CAP in the Indian Ocean region. There are only 2 studies concerning Madagascar but none concerning Mauritius or the Comoros archipelago (Razanajatovo NH, Guillebaud J, Harimanana A, Rajatonirina S, Ratsima EH, Andrianirina ZZ, Rakotoariniaina H, Andriatahina T, Orelle A, Ratovoson R, Irinantenaina J, Rakotonanahary DA, Ramparany L, Randrianirina F, Richard V, Heraud JM. Epidemiology of severe acute respiratory infections from hospital-based surveillance in Madagascar, November 2010 to July 2013. PLoS One. 2018 Nov 21;13(11):e0205124. doi: 10.1371/journal.pone.0205124. eCollection 2018) ([J L Rakotoson](https://pubmed.ncbi.nlm.nih.gov/?sort=date&term=Rakotoson+JL&cauthor_id=20337118), [J R Rakotomizao](https://pubmed.ncbi.nlm.nih.gov/?sort=date&term=Rakotomizao+JR&cauthor_id=20337118), [A C F Andrianarisoa](https://pubmed.ncbi.nlm.nih.gov/?sort=date&term=Andrianarisoa+AC&cauthor_id=20337118). Acute community acquired pneumonia: 96 cases in Madagascar . Med Trop (Mars) 2010 Feb;70(1):62-4).

It has been added in the revised manuscript (line 217).

3. ***Line 79, 81 &118, change “legionella” to “*Legionella*” since genus used alone should be capitalized and italicized.***

*W*e apologize, it has been corrected in the revised manuscript

4. ***Line 82,153, 194, 233 & 301,*Streptococcus pneumonia *should be* “S. pneumoniae”**

We apologize, it has been corrected in the revised manuscript

5. **Line 116 – 117, change “Chlamydia pneumonia, Mycoplasma pneumonia, Legionella spp, Haemophilus influenza, Streptococcus pneumoniae and Bordetella (para) pertussis.” to “Chlamydia pneumoniae, Mycoplasma pneumoniae, Legionella spp, Haemophilus influenzae, S. pneumoniae, Bordetella pertussis, and B. prarpertussis.”**

We apologize, it has been corrected in the revised manuscript

6. Line 142, please rephrase “…(incidence of 22 per 100,000 person- years)” for clarity.  Did the author mean 22 per 100,000 person per year?

We agree with reviewer comment it has been modified in the revised manuscript

7. ***Line 154, Staphylococcus should be italicized*.**
8. **Line 155, “Haemophilus influenza” should be “H. influenzae”**
9. **Line 155 – 156, “Legionella pneumophila” *should be* “L. pneumophila”**
10. **Table 3, “Haemophilus influenza” *should be* “Haemophilus influenzae”**

We apologize, it has been corrected in the revised manuscript

11. ***Line 193 – 195, it is not clear about the correlation between rainfall and CAP, please expand the explanation.***

We agree with reviewer comment. Several studies find an association between seasons and the occurrence of CAP but the physio-pathological explanation of this association remains to be established. This explanatory paragraph was removed because it was unclear

12. ***Line 200 – 206, maybe I missed something, I am not clear on the relevance of the risk factors for influenza with CAP?  How about risk factor associated with* S. pneumoniae *or CAP in general?***

We agree with reviewer comment, this analysis has been removed from the revised manuscript. Risk factors for developing CAP could not be assessed because there was no control group (without CAP).

13. ***Line 220 – 222, please clarify the relevance of this statement.***

We agree with reviewer comment this section has been removed from the revised manuscript

14. **Line 248, change “K. pneumoniae” to “Klebsiella pneumonia” since this is the first time Klebsiella was mentioned.**
15. **Line 274, Actinobacter should be italicized.**
16. **Line 285, “Staphylococcus aureus” should be “S. aureus”**

We apologize, it has been corrected in the revised manuscript

***Reviewer #1:***

***Thank you for sending me this manuscript for review. The investigators studied clinical and microbiological characteristics of patients with severe pneumonia who were admitted to the ICUs. The manuscript is of interest, however, several points need to be highlighted and revised as follow.***

***Abstract
Editing error: “causatives”***

We agree with reviewer comment, it has been corrected in the revised manuscript.

***Conclusions are not based on the results. Also, the recommendation of cephalosporin and oseltamivir needs revision to reflect the study findings and may lead to increased antimicrobial resistance***.

We agree with reviewer comment, it has been corrected in the revised manuscript.

***Introduction
Some general statements require evidence and to be specific, for example:
Initiate antibiotic therapy against all strains of S pneumonia and Legionella. This is a general statement that needs to be more specific on the empiric use of antimicrobials.
We agree with reviewer suggestion, it has been added in the revised manuscript***

We agree with reviewer suggestion, it has been précised in the revised manuscript:i.e. a combination therapy with cephalosporin and macrolide or monotherapy with respiratory fluoroquinolone (83-85).

***Methods
The study was conducted on ICU admitted patients, and they may not represent the study objectives rather than only severe cases that required ICU admission. So, study objectives need to be changed accordingly.***

We agree with reviewer comment, it has been modified in the revised manuscript that it was all severe case of CAP **hospitalized in ICU (line 91, 105)**.

***It’s not clear too, how data were collected, was it a retrospective chart review?***

It was a chart review of all patients hospitalized in ICU for severe CAP. It has been precized in the revised manuscript (line 104).

**Statistical analysis**

**Line 5: First time mentioned abbreviation, the full name should be provided.
Also, the statement is not clear: “Risk factors of influenza PAC in bivariate analysis with P<0.1 were entered into a multivariate logistic regression analysis using backward selection with P <0.05”.**

Has suggested by the Editor, the section on risk factors of Influenza CAP with multivariate analysis has been removed from the revised manuscript.

***The reported incidence in the results section was different from the abstract and discussion, should be revised.***

We apologize, it has been corrected in the revised manuscript

***Table (1), abscess was mentioned, what does it refer to what type of abscess and which site?***

It was pulmonary abscess, it has been added in the revised manuscript

***Table (1), duration between symptoms and ICU admission, was it days?***

It was days, it has been précised in the revised manuscript

***Table (1), what does it mean of reporting the p-value as zero? (for influenza-like illness)***

We apologize, this was an error, and these are P < 0.001. This has been corrected in the revised manuscript.

***Table (1), how hazardous alcohol use was assessed, what definition was used, and how data were collected?***

We have used The Alcohol Use Disorders Identiﬁcation Test Consumption (AUDIT-C) with a with a threshold value of 4 for men and 3 for women ([Bradley](https://pubmed.ncbi.nlm.nih.gov/?sort=date&term=Bradley+KA&cauthor_id=17451397) KA,  [DeBenedetti](https://pubmed.ncbi.nlm.nih.gov/?sort=date&term=DeBenedetti+AF&cauthor_id=17451397) AF,  [Volk](https://pubmed.ncbi.nlm.nih.gov/?sort=date&term=Volk+RJ&cauthor_id=17451397) RJ,  [Williams](https://pubmed.ncbi.nlm.nih.gov/?sort=date&term=Williams+EC&cauthor_id=17451397) EC,  [Frank](https://pubmed.ncbi.nlm.nih.gov/?sort=date&term=Frank+D&cauthor_id=17451397) D,  [Kivlahan](https://pubmed.ncbi.nlm.nih.gov/?sort=date&term=Kivlahan+DR&cauthor_id=17451397) DR. AUDIT-C as a brief screen for alcohol misuse in primary care. Alcohol Clin Exp Res 2007 Jul;31(7):1208-17. doi: 10.1111/j.1530-0277.2007.00403.x). It has been added in the revised manuscript.

***Table (1), what does footnote 2 refer to?***

It was a spelling error and it has been removed from the revised manuscript

***Did the investigators collect any vaccination histories on influenza or pneumococcal vaccines among the study participants?***

We apologize we dot not have this data

***Table (3), footnote … “…. during flu cases” need revision.
What is “flu syndrome” mentioned in line 201?***

We apologize during flu cases has been replaced by “Influenza CAP”. “Flu syndrome” was “Influenza-like illness” but it has been removed from the revised manuscript has it was in the multivariate analysis section.

***Higher leukocyte counts mentioned in line 204 is not a risk for severe influenza as per the odds ratio presented.***

We agree with reviewer comment. Moreover, it has been removed from the revised manuscript has it was in the multivariate analysis.

***Line 212 need revision***

We apologize, the line has been revised

***Discussion
The second highest cause of mortality and years of life lost. Was there a difference between mortality and years of life lost?***

We have deleted the second part of the sentence

**in-ICU mortality in this study was lower than previously reported. Any specific reasons or contributing factors to be discussed?**

It has been reported that mortality for CAP could reach 50% in patients hospitalized in ICU. Nevertheless, mortality in recent studies that evaluated severe CAP in-ICU patients was similar to that in our study: 22% in the study by Ferrer et al ([Ferrer](https://pubmed.ncbi.nlm.nih.gov/?sort=date&term=Ferrer+M&cauthor_id=29370285) M, [Travierso](https://pubmed.ncbi.nlm.nih.gov/?sort=date&term=Travierso+C&cauthor_id=29370285) C, [Cilloniz](https://pubmed.ncbi.nlm.nih.gov/?sort=date&term=Cilloniz+C&cauthor_id=29370285) C, [Gabarrus](https://pubmed.ncbi.nlm.nih.gov/?sort=date&term=Gabarrus+A&cauthor_id=29370285) A, [Ranzani](https://pubmed.ncbi.nlm.nih.gov/?sort=date&term=Ranzani+OT&cauthor_id=29370285) OT, [Polverino](https://pubmed.ncbi.nlm.nih.gov/?sort=date&term=Polverino+E&cauthor_id=29370285) E, [Liapikou](https://pubmed.ncbi.nlm.nih.gov/?sort=date&term=Liapikou+A&cauthor_id=29370285) A, [Blasi](https://pubmed.ncbi.nlm.nih.gov/?sort=date&term=Blasi+F&cauthor_id=29370285) F, [Torres](https://pubmed.ncbi.nlm.nih.gov/?sort=date&term=Torres+A&cauthor_id=29370285) A. Severe community-acquired pneumonia: Characteristics and prognostic factors in ventilated and non-ventilated patients PLoS One 2018 Jan 25;13(1):e0191721. doi: 10.1371/journal.pone.0191721. eCollection 2018) and 27% in the study by Cavallazzi et al ([Cavallazzi](https://pubmed.ncbi.nlm.nih.gov/?sort=date&term=Cavallazzi+R&cauthor_id=32298730) R, [Furmanek](https://pubmed.ncbi.nlm.nih.gov/?sort=date&term=Furmanek+S&cauthor_id=32298730) S,  [Arnold](https://pubmed.ncbi.nlm.nih.gov/?sort=date&term=Arnold+FW&cauthor_id=32298730) FW, [Beavin](https://pubmed.ncbi.nlm.nih.gov/?sort=date&term=Beavin+LA&cauthor_id=32298730) LA, [Wunderink](https://pubmed.ncbi.nlm.nih.gov/?sort=date&term=Wunderink+RG&cauthor_id=32298730) RG, [Niederman](https://pubmed.ncbi.nlm.nih.gov/?sort=date&term=Niederman+MS&cauthor_id=32298730) MS, [Ramirez](https://pubmed.ncbi.nlm.nih.gov/?sort=date&term=Ramirez+JA&cauthor_id=32298730) JA. The Burden of Community-Acquired Pneumonia Requiring Admission to ICU in the United States. Chest 2020 Sep;158(3):1008-1016. doi: 10.1016/j.chest.2020.03.051. It has been added in the discussion of the revised manuscript (line 271-275)

***Editing, line 253, “….. we find” should “found”***

We apologize it has been corrected

***What about the impact of empirical use of antimicrobials on the detection of organisms that have been reported in other reports.***

The use of empirical antibiotics could theoretically have an impact on deep respiratory samples. But to our knowledge there is no study on the impact of one dose of antibiotic on the positivity of respiratory samples concerning CAP***.***

***The investigators included a paragraph in the discussion about climate change and influenza types, although they didn’t provide a specific discussion or explored the relevance to the study.***

We agree with reviewer suggestion, has also suggested by the Editor this section has been has been removed from the revised manuscript. Several studies find an association between seasons and the occurrence of CAP but the physio-pathological explanation of this association remains to be established. This explanatory paragraph was removed because it was unclear.

***Conclusions should be revised to reflect the study findings.***

We agree with reviewer suggestion, the conclusions have been modified.

***Also, the recommendation of probabilistic antimicrobials and oseltamivir to every patient should be revised taking into consideration the potential risk for antimicrobial resistance and definition of severe pneumonia that may not be specific to be applicable in several care settings.***

We agree with reviewer comment, we have removed from the revised manuscript the text concerning the treatment by oseltamivir.

***Figure (2), worst survival was observed for cases with S pneumonia, this needs further discussion.***

In the present study, the mortality rate in the subgroup of patients with S. pneumonia CAP was similar to that reported by Dupuis *et al.* They found that in-hospital mortality among the 1665 patients who required ICU admission for pneumococcal CAP was 22.8% on day 28 ([Dupuis](https://pubmed.ncbi.nlm.nih.gov/?sort=date&term=Dupuis+C&cauthor_id=33423691) C , [Sabra](https://pubmed.ncbi.nlm.nih.gov/?sort=date&term=Sabra+A&cauthor_id=33423691) A, [Patrier](https://pubmed.ncbi.nlm.nih.gov/?sort=date&term=Patrier+J&cauthor_id=33423691) J, [Chaize](https://pubmed.ncbi.nlm.nih.gov/?sort=date&term=Chaize+G&cauthor_id=33423691) G, [Saighi](https://pubmed.ncbi.nlm.nih.gov/?sort=date&term=Saighi+A&cauthor_id=33423691) A, [Féger](https://pubmed.ncbi.nlm.nih.gov/?sort=date&term=Féger+C&cauthor_id=33423691) C, [Vainchtock](https://pubmed.ncbi.nlm.nih.gov/?sort=date&term=Vainchtock+A&cauthor_id=33423691) A, [Gaillat](https://pubmed.ncbi.nlm.nih.gov/?sort=date&term=Gaillat+J&cauthor_id=33423691) J, [Timsit](https://pubmed.ncbi.nlm.nih.gov/?sort=date&term=Timsit+JF&cauthor_id=33423691) JF. Burden of pneumococcal pneumonia requiring ICU admission in France: 1-year prognosis, resources use, and costs. Crit Care 2021 Jan 10;25(1):24. doi: 10.1186/s13054-020-03442-z.). It has been added in the discussion section of the revised manuscript line 271-275).

***Figure (1) (which is also written as figure (2)), about rainfall and influenza, showed three peaks of rainfall but not clear of an association with the occurrence of influenza or pneumococcal infection, more elaboration in this regard is required.***

There is an association between the dry season and the occurrence of influenza CAP. This association was not found with *S. pneumoniae*. Several studies find an association between seasons and the occurrence of Influenza CAP but the physio-pathological explanation of this association remains to be established. This explanatory paragraph was removed because it was unclear.

***Reviewer #2: Severe community-acquired pneumonia in Reunion Island: Epidemiological, clinical and microbiological characteristics, 2016-2018 (Manuscript No. PONE-D-21-05049)
In their manuscript, de Mangou et al. present valuable data derived from a retrospective cohort of patients admitted and diagnosed with severe CAP at Reunion Island between 2016 and 2018. However, significant scarcities of methodology detailed below could be identified in the manuscript. Also, the main study results might be of more interest to the national readers of Reunion Island. In all, the Methods section needs rewriting by addition of more explicit details from the study protocol before the review process could be continued any further.
Remarks on methodology:***

***1. A major issue is that CAP was not defined properly (only criteria of severe CAP were stated), and details were not given on how cases of CAP were ascertained. A brief summary of in-hospital algorithms should be added to the manuscript. Also, did all the patients receive the same diagnostic (eg. imaging) and microbiological studies before the final diagnosis of CAP was arrived at***?

We have better redefined our diagnosis criteria for CAP in the material and methods section (line XX-XX). Data were collected using a chart review of all patients hospitalized in ICU for severe CAP. It has been also precized in the revised manuscript (108-112).

***2. What is a cytobacteriological examination? This needs further explaining.***

We apologize, the sentence has been reworded. Bacteriological examination of respiratory samples were always performed (sputum samples on non-intubated patients and tracheal or bronchoalveolar sputum on intubated patients (122).

***3. Did the patients have blood cultures taken?***

Yes, all patients had blood cultures. It has been précised in the revised manuscript (line 122).

***4. Are all patients with severe CAP hospitalized at the ICU ward at Reunion Island?***

It has been précised that it was all patients with severe CAP and hospitalized in ICU that have been evaluated. Indeed, some patients are not admitted to the intensive care unit because of ethical considerations

**5. How were eligible patients screened and included in the final cohort?**

We performed a retrospective **chart** review of all patients diagnosed with severe CAP. It has been précised in the revised manuscript (line 104).

***6. Was any patient follow-up seeked during the study period? If yes, how?***

Follow up was seeked during in-hospital hospitalization on chart patients

***7. How was data collection executed? What was the data source? How were missing data handled?***

Data collection was retrospectively collected on patient charts. And a column with the item missing data has been added in the tables.

***8. The primary outcome should be defined by a hard clinical end-point, eg. in-hospital all-cause mortality at day-14 or alike. "Clinical and microbiological characteristics" are not endpoints – they are characteristics.***

We agree with reviewer suggestion the primary outcome has been modified in the revised manuscript (line 137)

***9. There is a contradiction in the description of statistical methods: an univariate pre-screening was done (line 133), but after that, only the "most clinically relevant factors" were entered into multivariate analysis (line 135)? This needs to be elaborated.***

We agree with reviewer comment, has suggested by the editor but the multivariate analysis has been removed from the revised manuscript

***10. Adherence to the STROBE Statement is highly encouraged during rewriting.***

We agree with reviewer comment, adherence to the STROBE checklist has been checked in the revised manuscript (missing data in tables…) and a statement has been added (101).

***11. I suggest using the term bronchoalveolar lavage instead of bronchoalveolar sputum.***

We apologize, it has been corrected in the revised manuscript

***12. I suggest that the term "adult patients" should be emphasized.***

We agree with reviewer comment, it has been specified in the revised manuscript (104)

***13. The name of the American Thoracic Society should be corrected.***

We apologize, it has been corrected in the revised manuscript
