# Supplementary material for: Severe community-acquired pneumonia in Reunion Island: Epidemiological, clinical, and microbiological characteristics, 2016–2018
Source: PLoS One. 2022 Apr 15;17(4):e0267184. doi: 10.1371/journal.pone.0267184 (PMC9012352; doi:10.1371/journal.pone.0267184)
Supplement: Attachment — Submitted filename: Response to reviewer_R2.docx [file pone.0267184.s004.docx]

**COMMENTS TO AUTHOR:**

***Reviewer #1: Thank you for revising your manuscript. However, there are some points that I think need a revision.***

1. ***Conclusion in the abstract and at end of the manuscript that melioidosis and Acinetobacter app should be considered in patients returning from Madagascar, this conclusion is based on only a few number of cases and shouldn't be generalized especially an adjusted regression analysis was not performed.***

We agree with reviewer comment, it has been deleted from the conclusion of the revised manuscript (line 68).

2. ***In the Materials and methods, the investigators mentioned that written information notice about the process of data collection was obtained, but they didn't report about severely ill patients and those on ventilators or not fully conscious, how that process was complete.***

We have clarified that in the revised manuscript. Information notice about the process of data collection was obtained from their legally authorized representative in case of patients unable to obtain the information (line 99).

3. ***In the Material and methods, third paragraph: a reference on the CAP clinical definition is required.***

We agree with reviewer comment, it has been added in the revised manuscript line 110).

4. ***In the results section: The acute physiology score was first mentioned with no explanation of its definition, components, or reference. The same was in Table1 for CURB-65.***

We agree with reviewer comment, references have been added in the revised manuscript (Lim WS, van der Eerden MM, Laing R, et al. (2003). ["Defining community acquired pneumonia severity on presentation to hospital: an international derivation and validation study"](https://www.ncbi.nlm.nih.gov/pmc/articles/PMC1746657)/ A New Simplified Acute Physiology Score (SAPS II) Based on a European/North American Multicenter Study. JAMA. 1993).

5. ***Table 2: Biological characteristics should be changed into laboratory findings.***

We apologize, it has been corrected in the revised manuscript

6. ***Discussion should highlight that severity of influenza-CAP may be associated with patients' underlying conditions not directly related to the influenza infection itself compared to other infections. This can be evidenced if a multivariate regression was performed.***

We agree with reviewer comment. We found that patients in the group with influenza CAP had higher mortality rate than patients with non-influenza CAP following univariate analysis. Nevertheless, patients with influenza CAP had more frequent comorbidities (age over 65 years old, asthma, diabetes mellitus…) and there is no multivariate analysis performed. It has been added in the revised manuscript (line 275).

7. ***Kruskal-Walis test was mentioned in the statistical analysis, although not shown or used in the results.***

We agree with reviewer comment. It has been deleted from the revised manuscript.

***Reviewer #2: In their revised manuscript, de Mangou et al. addressed all the methodological remarks. A reconsideration of publication could be recommended***.

Specific comments:

1. ***Line 78 – 80:  This statement needs reference(s).***

We agree with reviewer comment. References have been added in the revised manuscript

Microbial Etiology of Pneumonia: Epidemiology, Diagnosis and Resistance Patterns. Cilloniz et al. 2016

But clearly, it can’t be generalized because there are differences between causative organisms encountered in the tropics compared with temperate climates. Due to both environmental and socio- economic factors, diseases such as melioidosis, leptospirosis and tuberculosis are more widespread in the tropics. For example, observation of the aetiologies of community-acquired pneumonia (CAP) in an Asian outpatient setting showed that the most common isolates were Chlamydophila pneumoniae, followed by Mycoplasma pneumoniae and Streptococcus pneumoniae. Lim_et_al-2018-Respirology_pneumonia in the tropics.

2. ***Line 83 & 163 :  Change “S. pneumonia” to “S. pneumoniae”, “H. influenza” to “H. influenzae”.***

*W*e apologize, it has been corrected in the revised manuscript

3. ***Line 100:  this statement needs further clarification.***

We agree with reviewer comment, it has been clarified in the revised manuscript (line 98).

4. ***Line 122 – 124:  Needed to specify the methodology used? Culture or other methods?***

We agree with reviewer comment it has been specified in the revised manuscript that identification was performed with Gram staining followed by culturing with definite microorganism identification. Alternatively, identification was performed with matrix-assisted laser desorption ionization time-of-flight mass spectrometry.

5. ***Line 252:  Change “Acinetobacter” to “Acinetobacter”.***

*W*e apologize, it has been corrected in the revised manuscript

6. ***Discussion:  Use either oseltamivir or Oseltamivir:  Be consistent.***

*W*e apologize, it has been corrected in the revised manuscript
