# Supplementary material for: Severe community-acquired pneumonia in Reunion Island: Epidemiological, clinical, and microbiological characteristics, 2016–2018
Source: PLoS One. 2022 Apr 15;17(4):e0267184. doi: 10.1371/journal.pone.0267184 (PMC9012352; doi:10.1371/journal.pone.0267184)
Supplement: Attachment — Submitted filename: response_reviewer_R3.docx [file pone.0267184.s005.docx]

**COMMENTS TO AUTHOR:**

**Editor**

**We suggest you thoroughly copyedit your manuscript for language usage, spelling, and grammar. If you do not know anyone who can help you do this, you may wish to consider employing a professional scientific editing service.**

*As recommended, the manuscript has been revised by an English translator specialized in medical English (Ariane Dorval) and has been greatly improved*

**Specific comments:**

1. ***Line 79, change etc to etc*.**

It has been corrected

2. ***Line 83, change i.e.a to i.e. a***

It has been corrected

3. ***Line 96, change N° to No.***

It has been deleted

4. ***Line 98, delete "about the process of data collection"***

It has been deleted

5. ***Line 265, change S. pneumonia to S. pneumoniae***

It has been corrected

6. ***Line 276, change "...diabetes mellitus…" to "...diabetes mellitus, etc."***

It has been corrected

**Reviewers' comments:

Reviewer 1**

*No specific comment*
